# Up-regulation of casein kinase 1ε is involved in tau pathogenesis in Alzheimer’s disease

**DOI:** 10.1038/s41598-017-13791-5

**Published:** 2017-10-18

**Authors:** Caoyi Chen, Jianlan Gu, Gustavo Basurto-Islas, Nana Jin, Feng Wu, Cheng-Xin Gong, Khalid Iqbal, Fei Liu

**Affiliations:** 1 Key Laboratory of Neuroregeneration of Jiangsu Province and Ministry of Education of China, Co-innovation Center of Neuroregeneration, Nantong University, Nantong, Jiangsu 226001 P. R. China; 2 Department of Neurochemistry, New York State Institute for Basic Research in Developmental Disabilities, Staten Island, New York, 10314 USA; 3 Department of Genetics, School of Life Science, Nantong University, Nantong, Jiangsu 226001 P. R. China; 4 Present Address: Division of Sciences and Engineering, University of Guanajuato, León, Guanajuato Mexico

## Abstract

Hyperphosphorylation of tau and imbalanced expression of 3R-tau and 4R-tau as a result of dysregulation of tau exon 10 splicing are believed to be pivotal to the pathogenesis of tau pathology, but the molecular mechanism leading to the pathologic tau formation in Alzheimer’s disease (AD) brain is not fully understood. In the present study, we found that casein kinase 1ε (CK1ε) was increased significantly in AD brains. Overexpression of CK1ε in cultured cells led to increased tau phosphorylation at many sites. Moreover, we found that CK1ε suppressed tau exon 10 inclusion. Levels of CK1ε were positively correlated to tau phosphorylation, 3R-tau expression and tau pathology, and negatively correlated to 4R-tau in AD brains. Overexpression of CK1ε in the mouse hippocampus increased tau phosphorylation and impaired spontaneous alternation behavior. These data suggest that CK1ε is involved in the regulation of tau phosphorylation, the alternative splicing of tau exon 10, and cognitive performance. Up-regulation of CK1ε might contribute to tau pathology by hyperphosphorylating tau and by dysregulating the alternative splicing of tau exon 10 in AD.

## Introduction

Alzheimer’s disease (AD) is a slow neuroprogressive disorder histopathologically characterized by the presence of senile plaques and neurofibrillary tangles (NFT). Senile plaques are made of extracellular deposition of β-amyloid (Aβ) peptide derived from the cleavage of amyloid β-protein precursor (APP) by beta- and gamma-secretases. NFTs are composed of paired helical filaments (PHF), aggregated polymers of the abnormally hyperphosphorylated tau^1,2^. The density of NFTs directly correlates with the degree of dementia^3–6^.

Tau is the major neuronal microtubule associated protein. Its major biological function is to stimulate the assembly of microtubule (MT) and to stabilize the microtubule structure. In AD brain, tau is abnormally hyperphosphorylated, which leads to loss of its biological activity, gain of a toxic activity, and its aggregation into PHFs^7^. Thus, hyperphosphorylation of tau plays a pivotal role in tau pathogenesis in AD and other related neurodegenerative disorders called tauopathies.

Adult human brain expresses six isoforms of tau that are products of the alternative splicing of its pre-mRNA from a single gene. Tau exon 10 encodes the second MT-binding repeat, the alternative splicing of which generates tau isoforms with three or four MT-binding repeats, named 3R-taus or 4R-taus, respectively^8,9^. 3R-taus and 4R-taus differ in their biological function in polymerization and stabilization of neuronal microtubules as well as in their interactions with tau kinases^10–12^. The adult human brain expresses equal levels of 3R-taus and 4R-taus^13^. Several specific mutations of *tau* gene associated with frontotemporal dementias with Parkinsonism linked to chromosome 17 (FTDP-17) cause the alteration of ratio of 3R-tau and 4R-tau, but not tau primary sequence^14^. Thus, dysregulation of tau exon 10 splicing is sufficient to cause neurofibrillary degeneration^15,16^.

Casein kinase 1 epsilon (CK1ε) belongs to the CK1 family of ubiquitous serine/threonine–specific protein kinases. At least seven CK1 isoforms, named CK1α, -β, -γ1–3, -δ, and -ε, and their various splice variants have been described^17,18^. Each isoform consists of a highly conserved kinase domain followed by a highly variable C-terminal non-catalytic domain^18,19^. Members of the CK1 family are monomeric, constitutively active enzymes. By phosphorylating different substrates, such as cellular enzymes, transcriptional proteins, cytoskeletal and non-cytoskeletal proteins, viral oncogenes, and receptors, CK1 regulates diverse cellular processes, including circadian rhythms, cellular signaling, vesicular trafficking, cell division, and DNA repair pathways^19–22^.

CK1 has been suggested to have a role in tau pathology in AD brain^23^. The expression of CK1δ is markedly upregulated in AD brain regions-specifically^24^. The protein levels of CK1α, CK1δ and CK1ε are elevated 2.4-fold, 33-fold and 9-fold, respectively in AD hippocampus^25^. CK1δ phosphorylates tau at multiple sites and disrupts its binding to microtubules^23,26^. CK1ε shares 97% homology with CK1δ within its kinase domain and exhibits 53% homology within the C-terminal regulatory domain. However, the role of CK1ε in tau pathology is not well understood. In the present study, we found that the expression of CK1ε was increased dramatically in the frontal cortexes of the AD brains. Overexpression of CK1ε suppressed tau exon 10 inclusion and increased the phosphorylation of tau at multiple pathological epitopes in cultured cells and in mouse brain, which suggests that up-regulation of CK1ε may contribute to tau pathology in AD brain via dysregulation of phosphorylation and exon 10 splicing of tau and thus represents a promising target for therapeutic intervention.

## Results

### Expression of CK1ε is up-regulated in AD brain

**Figure 1:**
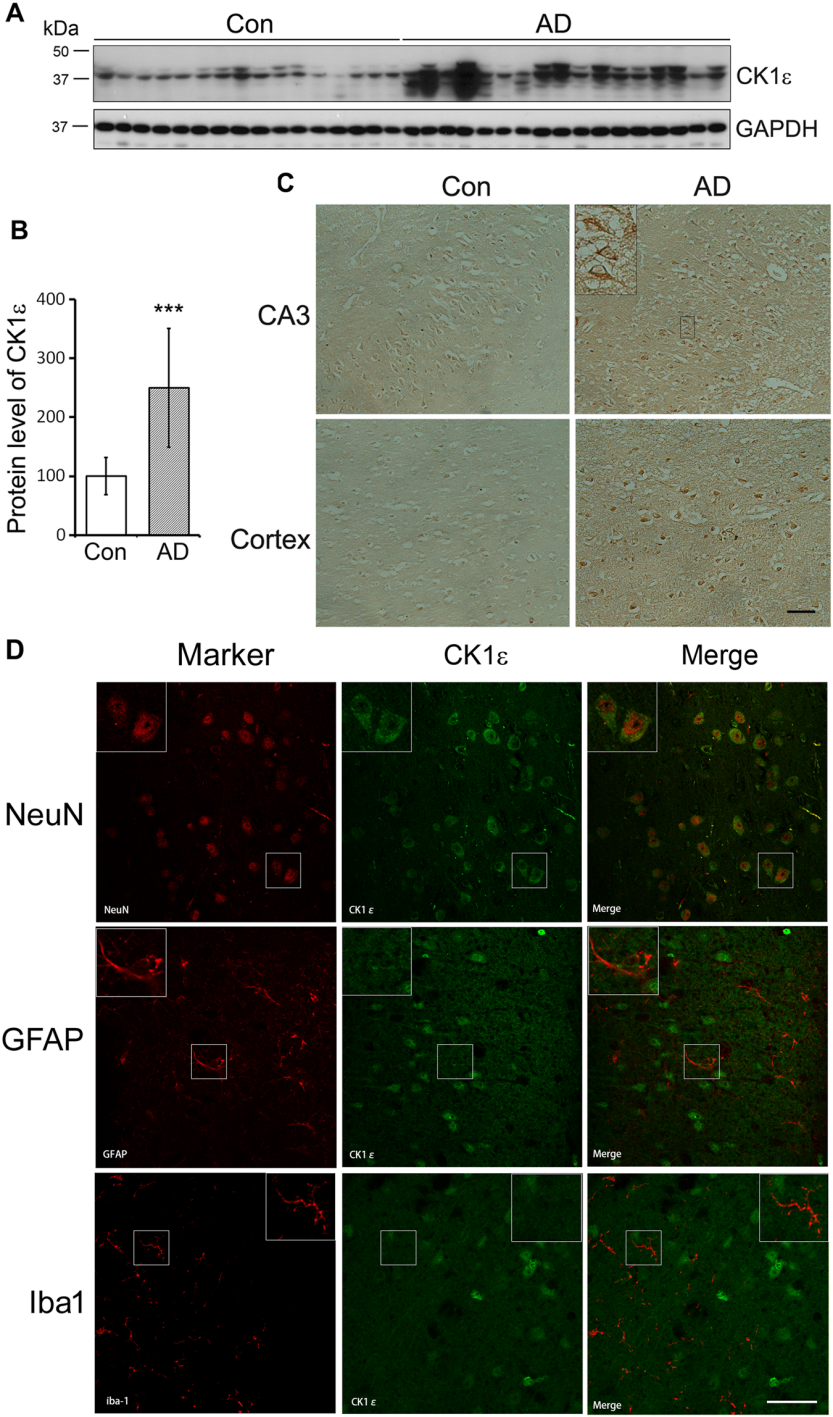
CK1ε is mainly expressed in neurons and elevated in AD brains. (**A**) CK1ε expression was increased in AD brain. Western blot analysis of the medial frontal cortex from 17 AD and 16 age- and postmortem delay-matched controls, developed with anti-CK1ε and anti-GAPDH, respectively. (**B**) The level of CK1ε was normalized with GAPDH after densitometric quantification. The data are presented as mean ± S.D.; ***p < 0.001. (**C**) Immunohistochemical staining shows the up-regulation of CK1ε in AD brains. Insert shows the expression of CK1ε in neuron. Scale bar = 200 μm. (**D**) Double immunofluorescence staining of CK1ε with NeuN (neuronal marker), GFAP (astraglia), or Iba1 (microglia) shows that CK1ε is mainly expressed in neurons in mouse brain. Scale bar = 50 μm.

To determine the role of CK1ε in tau pathogenesis, we first determined the levels of CK1ε in frontal cortices from 17 AD and 16 age- and postmortem delay-matched normal human brains by Western blots. We found that the expression of CK1ε was dramatically increased by 2.5 folds in AD brains as compared with controls (Fig. 1A,B). Immunohistochemical staining with anti-CK1ε also consistently showed that compared with control brains, the expression of CK-1ε was increased in AD in both the CA3 region of the hippocampus and cortex (Fig. 1C). By the morphology of the immunostaining, we found that CK1ε is mainly located in the neuronal cytoplasm (Fig. 1C, insert).

To study the expression of CK1ε in neuron, we performed double immunoflourecense in mouse brains by using antibodies against CK1ε and NeuN (neuronal marker), GFAP (astraglia marker), or Iba1 (microglia marker). We found that CK1ε was expressed in the cells with expression of NeuN, but not with GFAP or Iba1 (Fig. 1D), suggesting that CK1ε is mainly expressed in neurons.

### CK1ε interacts with tau

**Figure 2:**
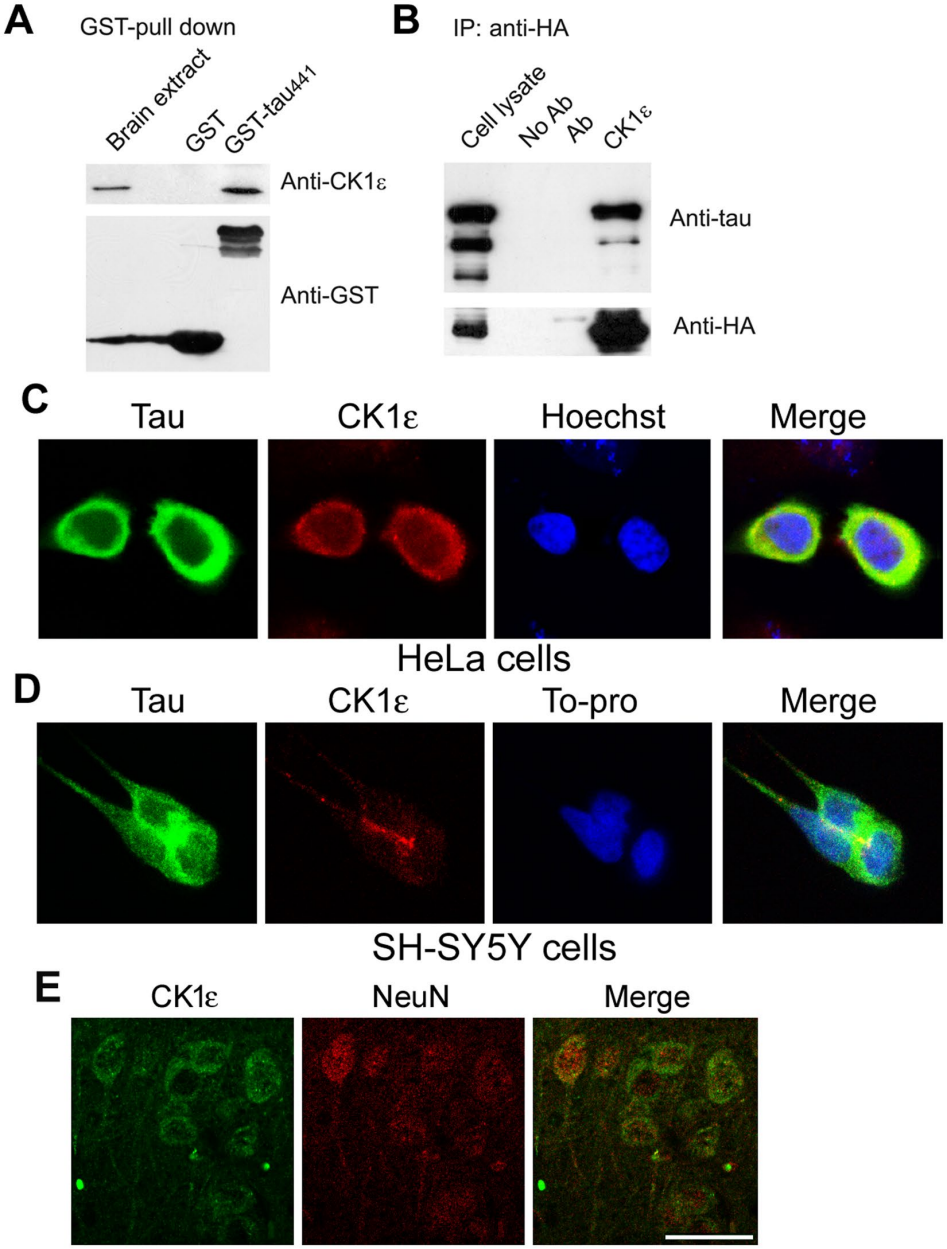
CK1ε interacts with tau. (**A**) CK1ε was pulled down by tau_441_. GST-tau_441_ or GST coupled onto glutathione-Sepharose was incubated with rat brain extract, and the bound proteins were analyzed by Western blots developed with anti-GST and anti-CK1ε. (**B**) tau_441_ was co-immunoprecipitated by CK1ε. CK1ε tagged with HA and tau_441_ were over-expressed in HEK-293T cells for 48 hrs. The cell extract was then immunoprecipitated with anti-HA, and the immunoprecipitated complexes (IP) were subjected to Western blots developed with antibodies indicated next to each blot. (**C**) CK1ε was co-localized with tau in HeLa cells. HeLa cells were co-transfected with tau-GFP and pCI-CK1ε-HA. At 48 hrs post-infection, cells were fixed and incubated with polyclonal anti-HA followed with Alexa 555 conjugated anti-rabbit IgG. Cells were examined for co-localization of tau and CK1ε using confocal microscopy; nuclei were identified by Hoechst 33342 staining. (**D**) CK1ε was co-localized with tau in SH-SY5Y cells. SH-SY5Y cells were immuno-stained with polyclonal anti-tau (R134d) and monoclonal anti-CK1ε and followed with Alexa Fluor 555- or Oregon Green 488-conjugated secondary antibodies from goat. (**E**) CK1ε was mainly expressed in neuronal cytoplasm. Mouse brain slides were double immunostained with anti-CK1ε and anti-NeuN and followed with fluorescence-conjugated second antibodies. Scale bar = 25 μm.

To determine the interaction between CK1ε and tau, we incubated glutathione S-transferase (GST)-tau coupled beads with rat brain extract for 2 hours at 4 °C. After extensive washing, the proteins pulled down with the beads were analyzed by Western blots. We found that CK1ε was pulled down by GST-tau, but not GST (Fig. 2A), suggesting that CK1ε interacts with tau.

To look for an interaction between CK1ε and tau in living cells, we performed co-immunoprecipitation experiments in HEK-293T cells co-overexpressing both proteins, CK1ε tagged with HA and tau_441_ fused with green fluorescence protein (GFP), GFP-tau_441_. CK1ε was immunoprecipitated with anti-HA and co-immunoprecipitated proteins were analyzed by Western blot with anti-tau. As shown in Fig. 2B, tau was detected in the co-immunoprecipitated proteins, supporting the possible physical interaction between CK1ε and tau.

To study CK1ε and tau interaction *in situ*, we co-expressed HA-tagged CK1ε and GFP-tau_441_ in HeLa cells and established their subcellular localization with confocal microscopy after immunofluorescent staining of CK1ε with anti-HA. We observed that both CK1ε (red fluorescence) and GFP-tau_441_ (green fluorescence) were mainly located in the cytoplasm. Then, we immuno-stained SH-SY5Y cells with monoclonal anti-CK1ε and polyclonal anti-tau. We also found that CK1ε (red) and tau (green) were co-localized in the cytoplasm (Fig. 2D). These data further support the interaction of CK1ε and tau in cultured cells.

Tau is a microtubule associated protein and is normally localized in the cytoplasm. However, in the brain tau cannot be immunohistochemically stained by using a standard method. To learn about the subcellular localization of CK1ε in mouse brain, we carried out the double immunoflourensencent staining in the mouse brain slides with anti-CK1ε and anti-NeuN, which is localized in the neuronal nucleus (Fig. 2E). We found that CK1ε was mainly localized in the cytoplasm (Fig. 2E), which is consistent with the above observation in Fig. 1C,D.

### Overexpression of CK1ε elevates tau phosphorylation in cultured cells

**Figure 3:**
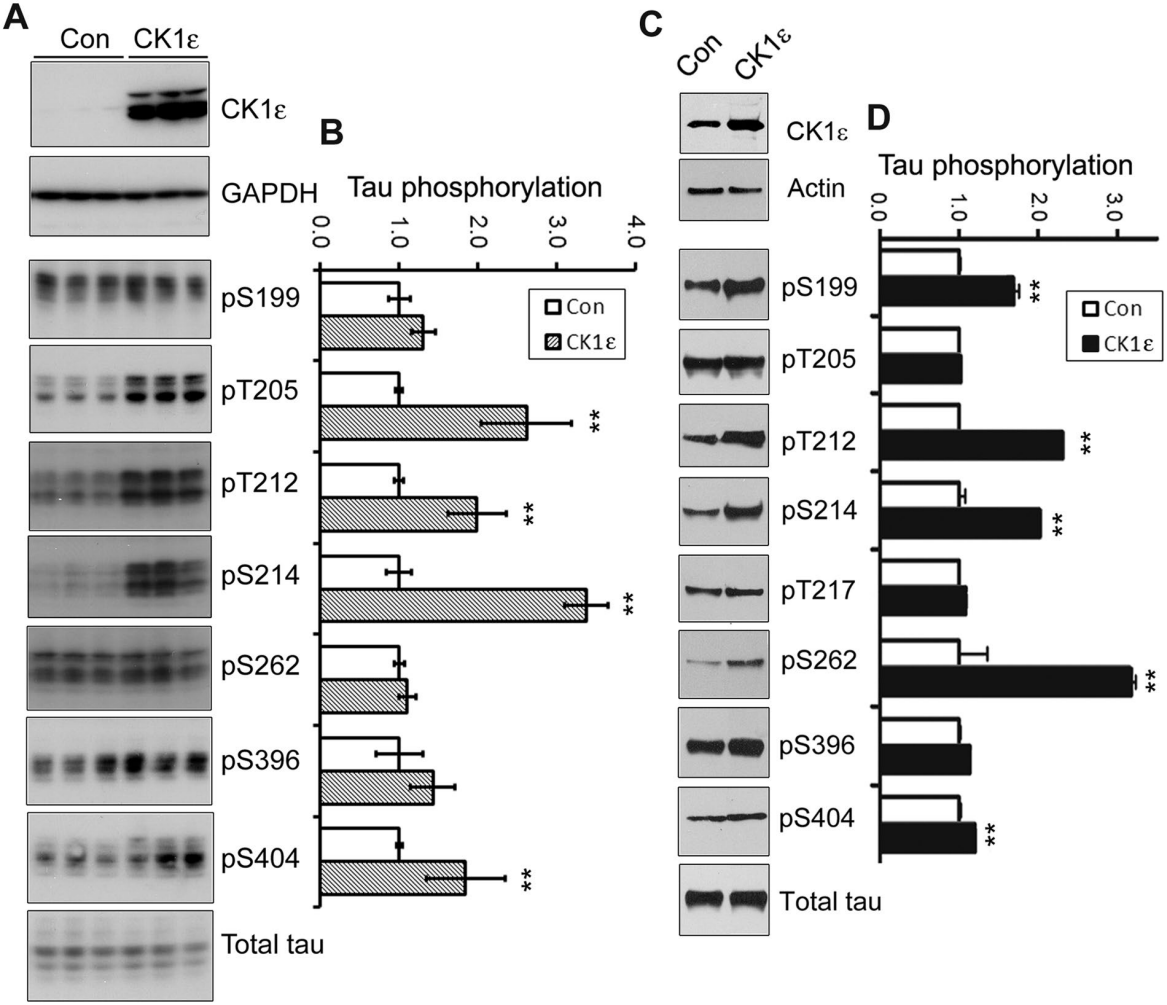
Overexpression of CK1ε increases phosphorylation of tau in cultured cells.(**A,**) (**B**) Overexpression of CK1ε increased tau phosphorylation. N2a cells were transiently transfected with pCI-CK1ε for 48 hrs. Phosphorylation levels of tau at multiple sites were analyzed by Western blots developed with CK1ε and phosphorylation dependent and site-specific tau antibodies and normalized by total tau level. (**C,D**) Overexpression of CK1ε increased tau phosphorylation in cultured neurons. Primary embryonic rat neurons 3 days in culture were infected with either lenti-control (Con) or lenti-CK1ε and analyzed 72 hrs after infection. CK1ε expression and tau phosphorylation at multiple sites were detected by Western blots developed with corresponding. The levels of tau phosphorylation at individual sites were normalized by total tau level. The data are presented as mean ± SD (n = 3–4); *p < 0.05; **p < 0.01 vs. control group.

Previously we found that CK1 phosphorylates tau effectively *in vitro* ^27^. To determine whether CK1ε modulates tau phosphorylation, we overexpressed CK1ε in N2a cells, and then determined phosphorylation of tau by Western blots developed with phosphorylation-dependent and site-specific tau antibodies. We found that phosphorylation levels of tau were markedly elevated at Thr205, Thr212, Ser214, and Ser404 in CK1ε overexpressing cells, compared with controls (Fig. 3). No significant changes were observed in total tau levels. Thus, CK1ε overexpression could significantly increase tau phosphorylation.

Next, we investigated whether CK1ε could phosphorylate tau in primary cortical neurons. We transduced with lentiviruses to express CK1ε in primary cortical neurons. Three days post-infection, the phosphorylation level of tau at many sites, including Ser199, Thr212, Ser214, Ser262, and Ser404 was significantly increased (Fig. 3C,D). This result confirms that CK1ε phosphorylates tau and increases tau phosphorylation at multiple sites in cortical neurons.

### Inhibition of CK1ε reduces tau phosphorylation

**Figure 4:**
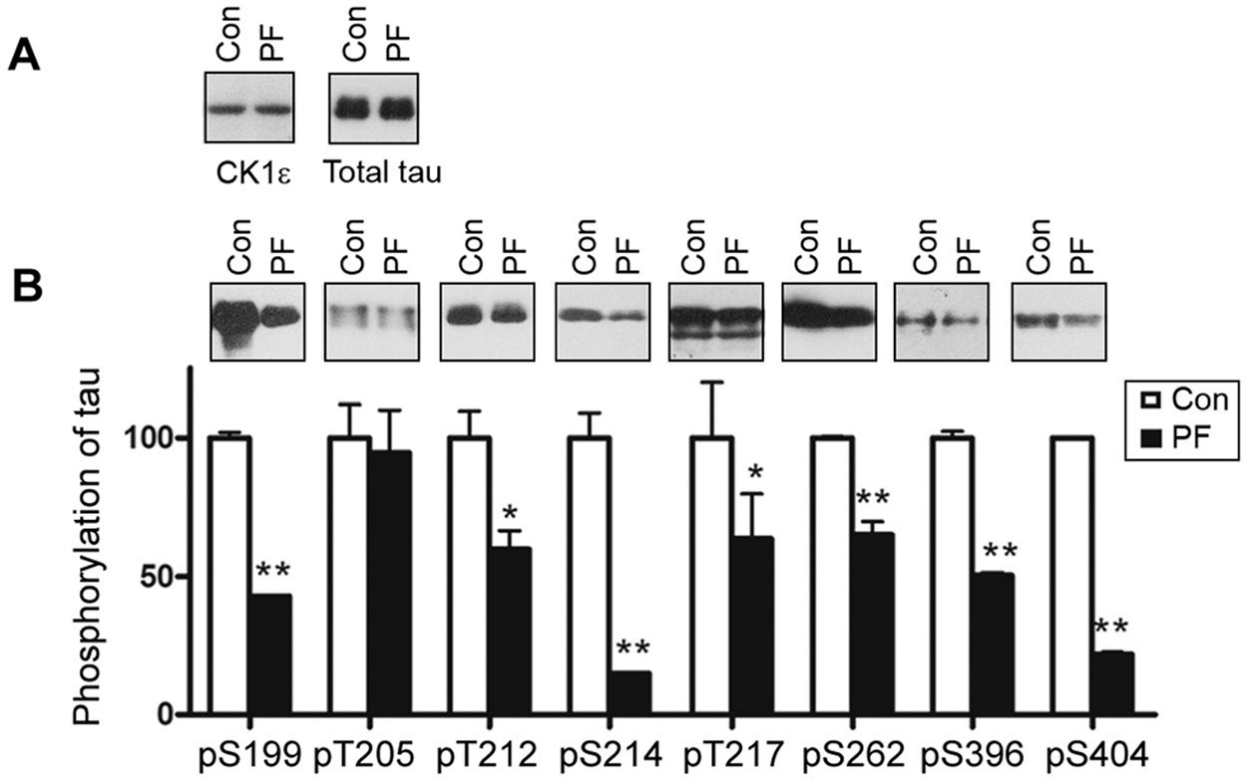
Inhibition of CK1ε suppresses tau phosphorylation. HEK-293FT cells were co-transfected with CK1ε and pCI/tau441 for 44 hrs and then treated with 1 μM PF4800567 for 4 hrs. CK1ε expression and phosphorylation of tau were determined by Western blots. Tau phosphorylation at individual sites was normalized by total tau level and presented as mean ± SD (n = 3); *p < 0.05; **p < 0.01.

PF4800567 is a selective inhibitor of CK1ε. To examine the effects of PF4800567 on tau phosphorylation, HEK-293FT cells were transiently co-transfected with pCI-tau_441_ and pCI-CK1ε or control pCI vector for 44 hours and then treated with 1 μM PF4800567 for 4 hours. The cell lysates were then subjected to Western blots to analyze tau phosphorylation at individual sites. We found that PF4800567 treatment did not affect CK1ε or tau expression (Fig. 4A). However, tau phosphorylation in the cells treated with PF4800567 was significantly decreased at Ser199, Thr212, Ser214, Ser217, Ser262, Ser396, and Ser404, indicating that selective CK1ε inhibitor PF4800567 treatment suppresses the phosphorylation of tau.

### Overexpression of CK1ε increases tau phosphorylation ***in vivo***

**Figure 5:**
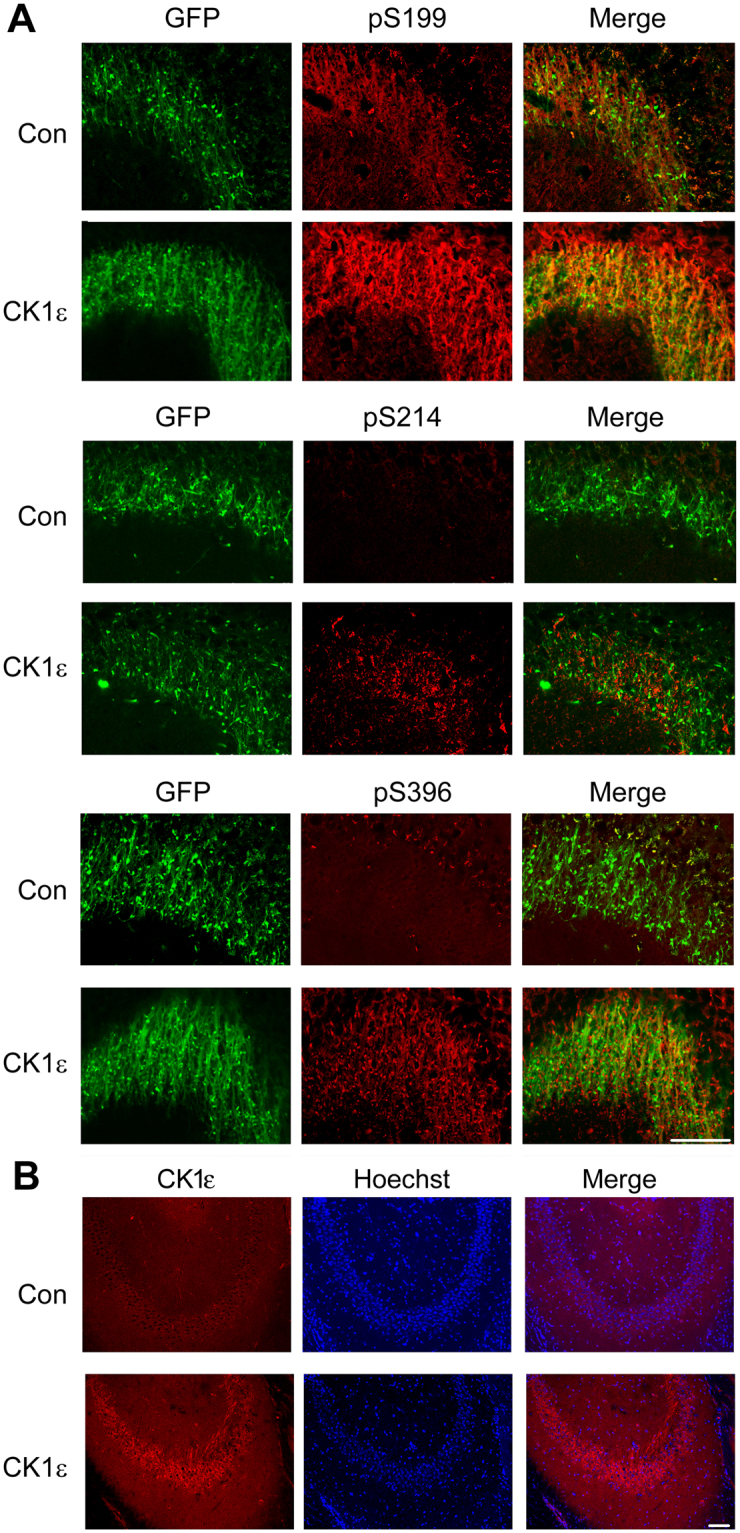
Overexpression of CK1ε increases phosphorylation of tau in the hippocampus. Lentiviral CK1ε or control virus was injected into the hippocampus of 3 months old tau_P301L_ mice. The mice were sacrificed for immunostaining with anti-phosphorylated tau or anti-CK1ε at 13 months of age. The representative images of GFP (green) and phosphorylated tau at Ser199, Ser396, and Ser214 (red) in mouse CA3 are shown in A and CK1ε is shown in B. Scale bar: 75 μm.

In order to investigate the role of CK1ε on tau phosphorylation *in vivo*, the lentivirus vector encoding GFP (Lenti-Con/GFP) or CK1ε and GFP (Lenti-CK1ε/GFP) was administered into the hippocampus of 3 month old tau_P301L_ mice. These animals were sacrificed for immunofluorescence analysis at the age of 13 months. We found the expression of GFP in hippocampus including CA3 area (Fig. 5A). To confirm lentiviral expression of CK1ε, we immunostained CK1ε with its antibodies and found that CK1ε immunoflourenscent staining was obviously stronger in the hippocampus with Lenti-CK1ε/GFP infection than with Lenti/GFP (Fig. 5B). Interestingly, direct intrahippocampal gene delivery of CK1ε indicated by GFP expression resulted in tau phosphorylation increase at site Ser396, Ser199, and Ser214 (Fig. 5A), suggesting that CK1ε overexpression in the CA3 area of the hippocampus induces tau phosphorylation.

### Level of CK1ε is correlated with hyperphosphorylation of tau

**Figure 6:**
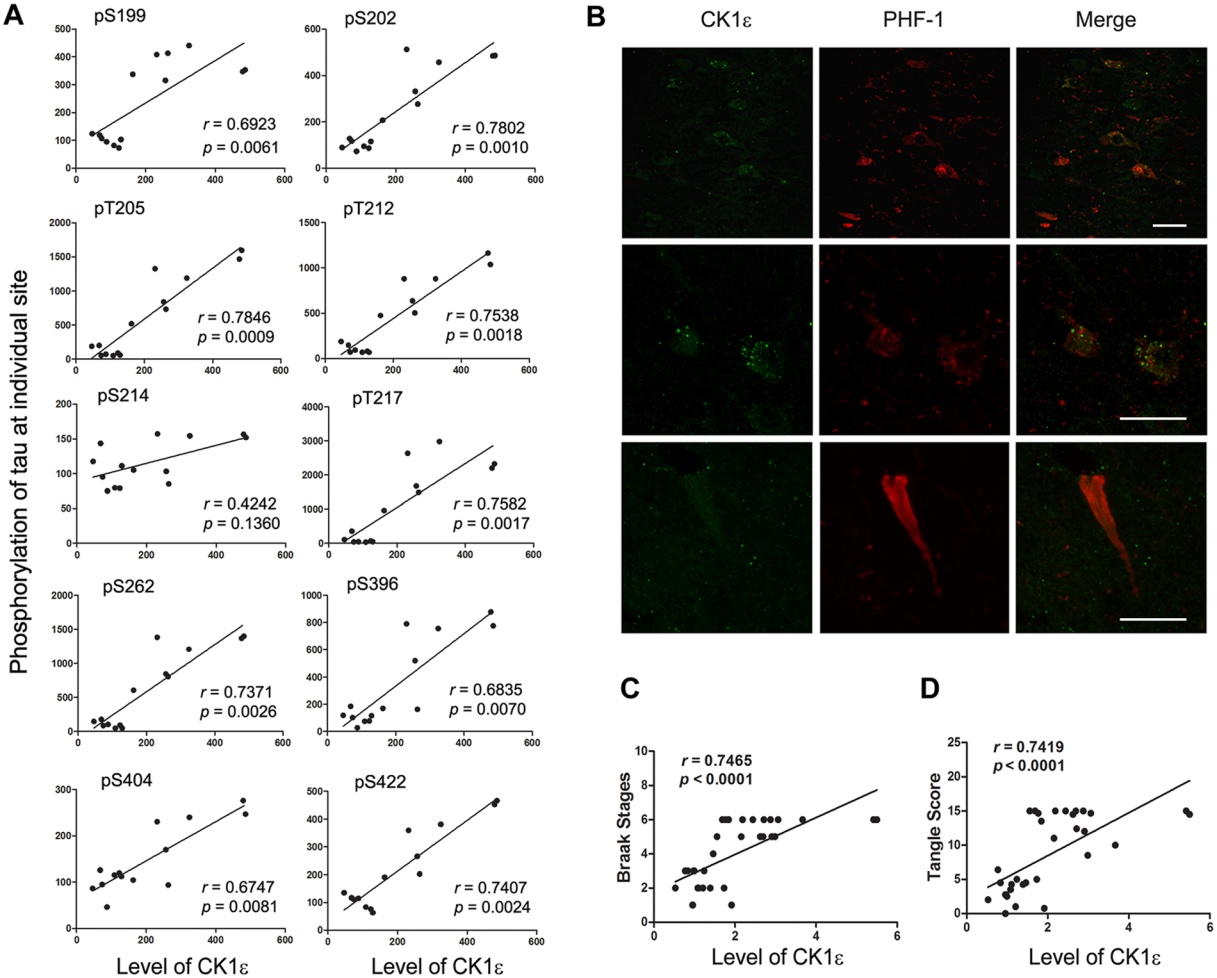
CK1ε is associated with hyperphosphorylation of tau. (**A**) Levels of CK1ε were correlated with tau phosphorylation in human brains. Levels of tau phosphorylation in the crude extracts of the frontal cortices from 7 AD and 7 control cases were determined by immuno-dot-blots. CK1ε levels in the brain homogenates were determined by Western blots. The level of tau phosphorylation at individual sites was plotted against the level of CK1ε. A linear regression line is shown in the graph. The Spearman correlation coefficient *r* was calculated and indicated in each panel. (**B**) CK1ε was co-localized with phosphorylated tau at Ser396/404. AD brain slides were double fluorescence stained with anti-CK1ε and PHF-1 and followed with Alexa Fluor 555-conjugated goat anti-mouse IgG and Oregon Green 488-conjugated goat anti-rabbit IgG. (**C,D**) CK1ε was correlated with tau pathology. The levels of CK1ε in brain homogenates of the frontal cortices form 17 AD and 16 control cases determined in Fig. 1A were plotted against the Braak Stages (**C**) and tangles scores (**D**). The Spearman correlation coefficient *r* was calculated.

To learn the role of CK1ε in tau phosphorylation in the human brain, we determined the levels of phosphorylation of tau at different sites by immune-dot blots^28^ and analyzed the linear regression between levels of CK1ε (Fig. 1A) and tau phosphorylation at individual sites in the frontal cortex of 7 AD and 7 control cases. We found that the levels of tau phosphorylation at the sites studied, except Ser214, was positively correlated with CK1ε level in the human brain (Fig. 6A), supporting the contribution of CK1ε to tau hyperphosphorylation in AD brain.

We confirmed the association of CK1ε with tau phosphorylation by double immunofluorescent staining of AD brain tissue sections with monoclonal PHF-1, an antibody against tau phosphorylated at Ser396/404, and polyclonal anti-CK1ε. We observed co-localization of CK1ε and PHF-1 in the neurons (Fig. 6B), suggesting the association between CK1ε and hyperphosphorylated tau. In addition, CK1ε was also found in tangle bearing neurons (Fig. 6B). These data further support the association of CK1ε with pathological tau.

NFTs are made up of hyperphosphorylated tau. Both Braak stage and CERAD Alzheimer’s Disease Criteria tangle score are methods used to classify the degree of tau pathology in AD^29,30^. Normal aged human brain has limited numbers of NFTs which is classified as Braak stage II–IV^31^. To determine the relationship of CK1ε to tangle score and Braak stage, we analyzed their correlation in 17 AD cases and 16 control cases and calculated the Spearman correlation coefficient *r*. We found that levels of CK1ε were positively correlated with the Braak stages and tangle scores in human brains (Fig. 6C,D). These results are consistent with the contribution of upregulated CK1ε to tau pathology in AD brain.

### CK1ε modulates the alternative splicing of tau exon 10

**Figure 7:**
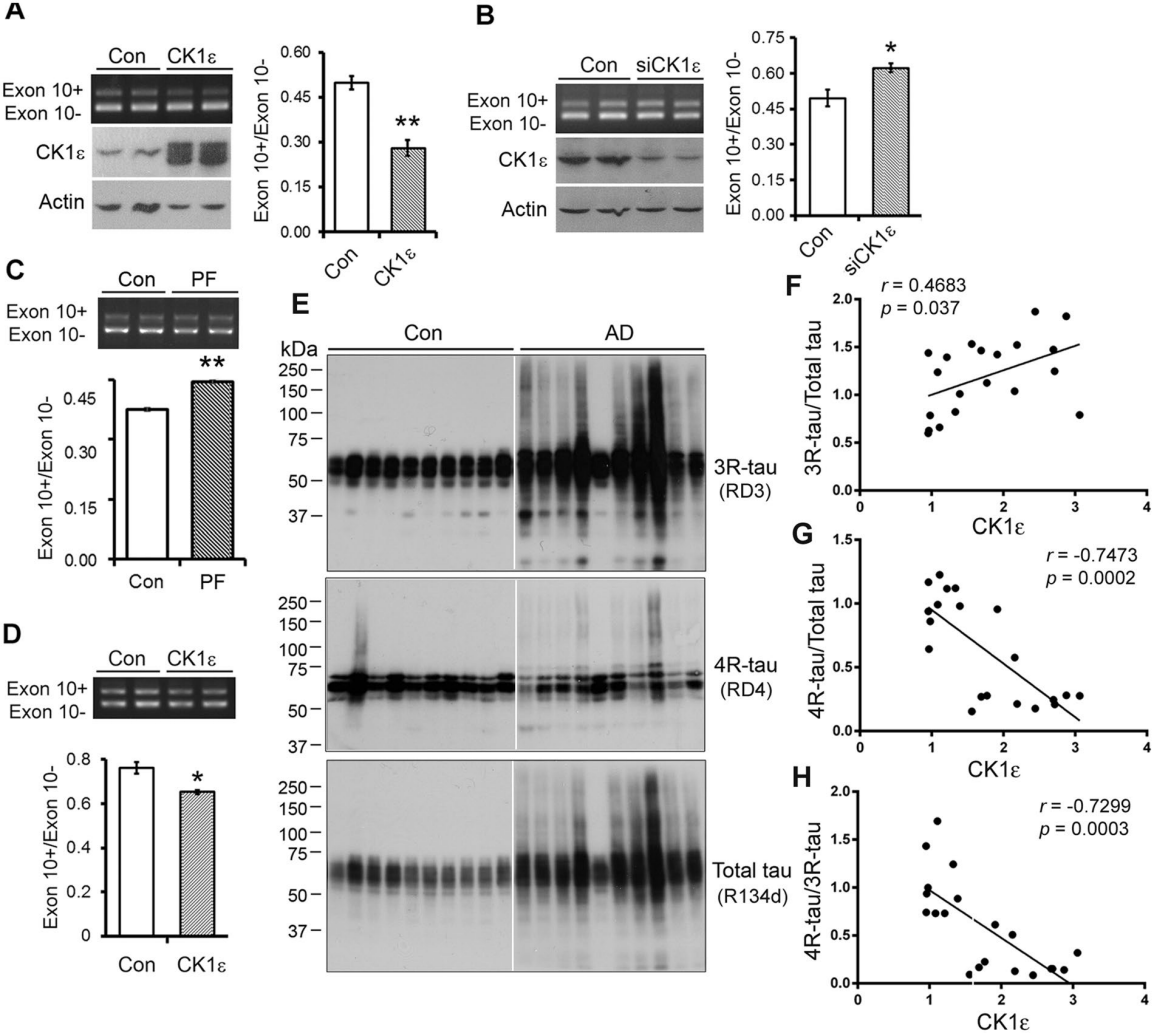
CK1ε suppresses tau exon 10 inclusion. (**A**) Overexpression of CK1ε suppressed tau exon 10 inclusion in HEK-293FT cells. HEK-293FT cells were co-transfected with pCI/SI9-LI10 mini-tau gene and CK1ε for 48 hrs and the splicing products of tau exon 10 were analyzed with RT-PCR and CK1ε expression was determined by Western blots. (**B**) Knock-down of CK1ε promoted tau exon 10 inclusion in HEK-293FT cells. HEK-293FT cells were co-transfected pCI/SI9-LI10 with siRNA of CK1ε. CK1ε expression was determined by Western blots and the splicing products of tau exon 10 were analyzed with RT-PCR 48 hrs after transfection. (**C**) Inhibition of CK1ε promoted tau exon 10 inclusion. HEK-293FT cells were transfected with pCI/SI-LI10 for 44 hrs and treated with 1 μM PF4800567 for 4 hrs. The splicing products of tau exon 10 were analyzed. (**D**) Overexpression of CK1ε suppressed tau exon 10 inclusion in cultured neurons. Primary embryonic rat neurons 3 days in culture were infected with either lenti-Con or lenti-CK1ε and analyzed 72 hrs after infection. The splicing products of tau exon 10 were analyzed with RT-PCR. (**E–H**) CK1ε may be correlated with the dysregulation of tau exon 10 splicing in AD brains. Levels of 3R-tau, 4R-tau and total tau in the crude extracts of the frontal cortices from AD and control cases was were determined by Western blots developed with RD3 (against 3R-tau), RD4 (against 4R-tau) and R134d (against total tau) (**E**). CK1ε level in the brain homogenates was determined by Western blots (Fig. 1A). The level of 3R-tau, 4R-tau or total tau was plotted against the level of CK1ε. A linear regression line is shown in the graph. The Pearson correlation coefficient r was calculated and indicated. Results are presented as mean ± S.D (n = 3); *p < 0.05; **p < 0.01.

Dysregulation of tau exon 10 causes neurofibrillary neurodegeneration^32^. To examine the effect of CK1ε on tau exon 10 splicing, we co-transfected tau mini-gene pCI/SI9-LI10 together with pCI-CK1ε into HEK-293FT cells and measured the alternative splicing products of tau exon 10 by RT–PCR. We found that over-expression of CK1ε markedly inhibited tau exon 10 inclusion (Fig. 7A), while knock-down of CK1ε by siRNA significantly enhanced tau exon 10 inclusion (Fig. 7B). Inhibition of CK1ε with PF4800567 also promoted tau exon 10 inclusion in pCI/SI9-LI10 transfected HEK-293FT cells (Fig. 7C). Similar inhibition of tau exon 10 inclusion was also observed in primary neuronal culture after infection with lentivirus carrying CK1ε (Fig. 7D). These results indicate that CK1ε regulates tau exon 10 splicing and suppresses tau exon 10 inclusion.

We previously reported that 3R-tau expression is increased in the AD brain^33^. To learn the relationship between CK1ε with 3R-tau and 4R-tau, we measured the 3R-tau, 4R-tau and total-tau in the homogenates of frontal cortices of 10 AD and 10 control brains by Western blots (Fig. 7E). Then we analyzed the linear regression between CK1ε and 3R-tau, 4R-tau and ratio of 4R-tau and 3R-tau. We found that the level of CK1ε was correlated with 3R-tau positively (Fig. 7F), but negatively correlated with 4R-tau (Fig. 7G) and ratio of 4R-tau/3R-tau (Fig. 7H) in human brains. These results suggest that CK1ε may play a role in the dysregulation of tau exon 10 splicing in AD brains.

### CK1ε overexpression in the hippocampus impairs spontaneous alternation behavior in mice

**Figure 8:**
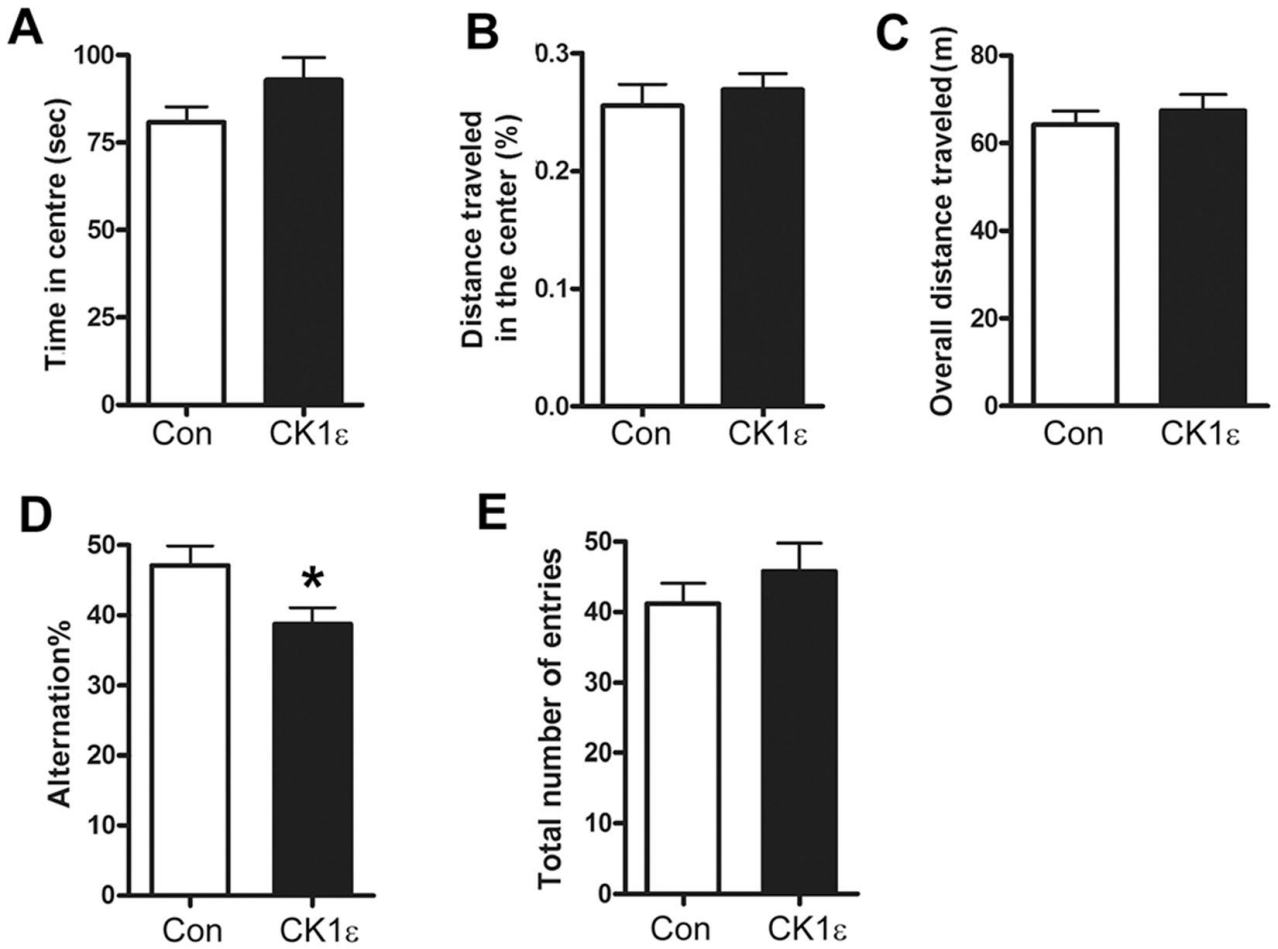
Hippocampal overexpression of CK1ε attenuates Y-maze spontaneous alternation. (**A–C**) Overexpression of CK1ε in mouse hippocampus did not affect motor activity/anxiety-related behavior. Tau_P301L_ mice were injected intra-hippocampally with lentiviral CK1ε or control virus at the age of 3 months and subjected to open filed test at 13 months. No difference in time spent in center region (**A**), the relative distance traveled the center zone (**B**), and overall distance traveled (**C**) between lentivrial CK1ε group and the control group was observed. (**D,E**) CK1ε overexpression in the hippocampus significantly decreased the percent of spontaneous alternations made by mice (**D**) and no significant difference was found in the total number of arm entries (**E**). Data are presented as mean ± SD (n = 10); *p < 0.05.

To investigate whether regulation of CK1ε in the hippocampus affects working memory *in vivo*, the tau_P301L_ mice with the lentivirus induced overexpression of CK1ε or GFP in the hippocampus were first subjected to motor activity-related behavior test of the control and lentiviral CK1ε mice by open field task at 13 months of age. We did not find differences in the amount of time spent in the open center zone (Fig. 8A), percentage of distance traveled in the center region (Fig. 8B) and the overall distance traveled (Fig. 8C), indicating similar general activity between lentiviral CK1ε injection mice and the controls. Thus, overexpression of CK1ε in mouse hippocampus did not significantly affect motor activity.

Following the open field test, the mice were tested for working memory by the Y maze spontaneous alternation assay. The spontaneous tendency to alternate free choices in entering the three arms of the Y maze is a measure of short-term working memory^34–36^. Lenti-CK1ε expressing transgenic mice displayed reduced alternation rates in this test compared to the control group (Fig. 8D), indicating impaired short-term working memory in these mice. Moreover, Lenti-CK1ε expressing mice did not show significant difference in the number of arm entries (Fig. 8E) compared to control mice. These data indicated no deficits in total activity level but a deficit in working memory in the lenti-CK1ε expressing mice.

## Discussion

Tau is abnormally hyperphosphorylated and accumulated as intraneuronal tangles in AD and related tauopathies^2,37,38^. Abnormal hyperphosphorylation of tau is believed to be critical to tau pathogenesis in AD. In the present study, we found that the level of CK1ε was elevated dramatically in AD brain. CK1ε interacted and was co-localized with tau. Overexpression of CK1ε in cultured N2a cells and primary cortical neurons increased phosphorylation of tau at several sites. In addition to phosphorylating tau, we found that CK1ε also suppressed tau exon 10 inclusion, leading to an increase in the ratio of 3R-tau:4R-tau. Elevation of CK1ε was found to correlate positively with tau phosphorylation, 3R-tau, and tau pathology presented by Braak Stages and Tangle Score in human brains. Lentiviral-induced overexpression of CK1ε in the hippocampus promoted tau phosphorylation and impaired working memory of the mice. Our findings suggest that upregulation of CK1ε in AD brain contributes to tau pathogenesis by phosphorylating tau and promoting 3R-tau expression.

CK1 purified from rat cerebellum phosphorylates tau effectively and induces AD-like epitopes much greater than other kinases *in vitro* ^27^. Truncated CK1δ, CK1δ_Δ317_, phosphorylates tau at ~30 sites^23^ and disrupts its binding to microtubules^26^. CK1ε shares 97% homology with CK1δ within their kinase domain and is predominantly expressed in the brain^39^. Here, we studied the role of full length CK1ε in tau phosphorylation in cultured cells and found that overexpression of CK1ε resulted in the elevation of tau phosphorylation at multiple sites. CK1 is a kinase that can be primed^40^ and can prime other kinases^41^. In addition, CK1 activity is regulated by its inhibitory autophosphorylation at the C-terminal domain^42,43^. Different types of cells express various types and/or levels of protein kinases and phosphatases. Thus, the phosphorylation of tau by overexpression of CK1ε may appear site-specifically in different types of cells.

Deregulation and mutations in the coding sequence of CK1 isoforms have been linked to several types of diseases, including neurodegenerative diseases^19^. It was reported that CK1ε expression was increased 9-fold in AD hippocampi relative to controls^25^. In the present study, we found that in the frontal cortex CK1ε protein level was increased 3-fold in AD compared with control cases. The differen cebetween these two studies may result from different brain regions in which the levels of tau pathology are different. Furthermore, the level of CK1ε was positively correlated with tau pathology, tau phosphorylation, Braak stages and tangle score positively, suggesting an association of overexpression of CK1ε with tau pathogenesis in the AD brain. Overexpression of CK1ε increased tau phosphorylation in cultured cells and *in vivo*. A previous study showed that in several tauopathies, including AD, CK1ε co-localized with neurofibrillary pathology^44^. The present study showed co-localization of CK1ε and PHF-1 in neurons. All of these findings indicate that the elevated CK1ε in the AD brain could contribute to tau hyperphosphrylation and pathology.

In addition to tau hyperphosphorylation, disturbance of 3R-tau/4R-tau ratio, which is generated by the alternative splicing of tau exon 10, also contributes to the development of tau pathology. Disruption of 3R-tau/4R-tau has been found in several human tauopathies, such as FTDP-17, Pick’s disease, progressive supranuclear palsy, corticobasal degeneration, and Down syndrome^15,45^. In the present study, we found that CK1ε can modulate tau exon 10 splicing and promote 3R-tau expression. Down-regulation of CK1ε by siRNA or by CK1ε inhibitor slightly suppressed tau exon 10 exclusion, suggesting CK1ε may play a certain role in tau exon 10 splicing. We recently found that CK1ε phosphorylates some splicing factors and regulates tau exon 10 splicing (data not shown). The expression of CK1ε in the AD brain is markedly increased and positively correlated with tau pathology. Thus, overexpression of CK1ε may contribute to tau pathogenesis.

Splicing factor ASF (alternative splicing factor) and SC35 play an important role in the tau exon 10 inclusion^46,47^. Their function is tightly regulated by phosphorylation. We previously found that dual-specificity tyrosine-phosphorylation regulated kinase 1 A (Dyrk1A) and protein kinase A (PKA) phosphorylated ASF and SC35 and were involved in the tau exon 10 splicing^46–48^. ASF and SC35 contain many non-proline directed serine or threonine residences. Thus, we speculate that CK1ε may regulate tau exon 10 splicing by phosphorylating the splicing factors.

Regulation of CK1 activity has hardly been studied. CK1 family members are monomeric, constitutively active enzymes, but CK1δ and CK1ε are regulated by inhibitory autophosphorylation at their C-termini^42,43^, which have been suggested to be acting as a pseudosubstrate that inhibits the enzyme activity. It has been reported that dephosphorylation of CK1ε C-terminal domain by activation of the serine/threonine phosphatase, calcineurin, also termed protein phosphatase (PP)2B, increases CK1ε kinase activity^49,50^. In the human brain, there are five highly expressed serine/threonine phosphatases, PP1, PP2A, PP2B, PP2C, and PP5. In AD brain, the total tau phosphatase activity is downregulated to 50%, which may underlie the abnormal hyperphosphorylation of tau. Kinetic analysis shows that PP1, PP2A, PP5 can regulate tau phosphorylation levels. As for PP2B, interestingly, it has a much lower affinity to tau protein and its activity was found to be increased by two-fold in AD brains compared with controls^51^. In this regard, one can speculate that PP2B up-regulation may dephosphorylate CK1ε inhibitory autophosphorylation domain and increase CK1ε activity, contributing to tau hyperphosphorylation. In our study, we failed to measure CK1ε activity in postmortem brain tissue due to technical difficulty.

In order to further investigate the role of CK1ε *in vivo*, we used a stereotactic injection of lentiviral CK1ε to the mouse hippocampus to increase CK1ε expression in the adult mouse brain. Behavioral analysis of mice injected with CK1ε revealed a significant reduction in Y-maze spontaneous alternation test but no deficits in locomotor function. Before alternation test, locomotor activity and anxiety level were assessed by open field tests. Both control and lentiviral CK1ε groups exhibited comparable total traveled distances and relative time and distance in the center of the open field, demonstrating no differences in exploratory drive or anxiety level. The results were further confirmed in elevated plus maze test (data not shown). No major differences were found in locomotor activity and anxiety like behavior between the control and lentiviral CK1ε groups. These data indicated that hippocampal CK1ε overexpression has no direct effect on locomotor activity, exploration motivation or anxiety level. Thus, the decreased alternation rate is not due to defects in locomotor level or motivation drive, suggesting CK1ε had a retardant effect on working memory. Since the locomotor activity was not impaired, we speculate that the reduced alternation rate depends on the hippocampal process rather than regulation of centers in the brain that control motor activity.

In summary, we found an increase in the expression of CK1ε in the AD brain and its direct correlation with tau pathology. Furthermore, we found that CK1ε was capable of promoting 3R-tau expression and phosphorylating tau at several AD sites in transfected cells and *in vivo* in tau_P301L_ mice. Over-expression of CK1ε in mice was found to result in the development of several characteristics of AD neuropathology including elevated tau phosphorylation and memory deficits. These findings strongly support that CK1ε is highly involved in tau pathogenesis; its deregulation may contribute to tau phosphorylation and the pathogenic processes in AD brain. These observations suggest the therapeutic potential of selective CK1ε inhibitors for the treatment of AD.

## Materials and Methods

### Human brain tissue

Medial frontal cortices from seventeen AD and sixteen age-matched normal cases used for this study (Table 1) were obtained from the Sun Health Research Institute Donation Program (Sun City, AZ); all cases were clinically and pathologically confirmed and stored at −70 °C until used. The use of autopsied frozen human brain tissue was in accordance with the National Institutes of Health guidelines and was approved by the IRB of New York State Institute for Basic Research in Developmental Disabilities (Protocol # 421). The research does not involve intervention or interaction with the individuals. The information is not individually identifiable.

**Table 1:** Basic information on Alzheimer’s disease (AD) and control (Con) cases used in this study.

Case	Age at death(year)	Gender	PMI^a^(h)	Braak stage^b^	Tangle scores^c^
AD1^e^	89	F	3.0	V	14.5
AD2^e^	80	F	2.25	VI	14.5
AD3^e^	85	F	1.66	V	12.0
AD4 ^e^	78	F	1.83	VI	15.0
AD5^e^	95	F	3.16	VI	10.0
AD6 ^e^	86	M	2.25	VI	13.5
AD7^e^	91	F	3.0	V	8.5
AD8^d^	83	F	3.0	VI	12.4
AD9^d^	74	M	2.75	VI	14.66
AD10^d^	79	F	1.5	VI	14.66
AD11^d^	73	F	2.0	V	15.00
AD12^d^	81	M	3.0	V	11.00
AD13^d^	76	M	2.33	VI	15.00
AD14^d^	72	M	2.5	VI	15.00
AD15^d^	74	F	2.83	VI	15.00
AD16^d^	76	M	4.0	V	15.00
AD17^d^	78	M	1.83	VI	15.00
mean ± S.D.	80.59 ± 6.70		2.52 ± 0.65		13.57 ± 2.05
Con1^d^	85	F	2.75	II	5.0
Con2^d^	82	F	2.0	II	4.25
Con3^d^	70	F	2.0	I	0.00
Con4^d^	73	M	2.0	III	2.75
Con5^d^	78	M	1.66	I	0.00
Con6^d^	80	M	3.25	I	2.75
Con7^d^	80	M	2.16	II	1.00
Con8^d^	83	F	3.25	I	0.75
Con9^d^	82	F	2.25	II	3.50
Con10^e^	85	M	2.5	II	4.25
Con11^e^	86	F	2.5	III	5.00
Con12^e^	81	M	2.75	III	6.41
Con13^e^	88	F	3.0	II	2.00
Con14^e^	90	F	3.0	III	4.50
Con15^e^	88	F	3.5	III	2.50
Con16^e^	88	F	3.0	IV	4.50
mean ± S.D.	82.44 ± 5.50		2.60 ± 0.55		3.07 ± 1.93

^a^PMI, postmortem interval.

^b^Neurofibrillary pathology was staged according to Braak and Braak (1995).

^c^Tangle score was a density estimate and was designated none, sparse, moderate, or frequent (0, 1, 2, or 3 for statistics), as defined according to CERADAD criteria. Five areas (frontal, temporal, parietal, hippocampal, and entorhinal) were examined, and the scores were added up for a maximum of 15.

^d^Cases were used for 3R-tau measurement by immuno-dot-blots.

^e^Cases were used for tau phosphorylation measurement by immuno-dot-blots.

### Animals

The transgenic FVB-tau_P301L_ mice, FVB-Tg(tetO-MAPT*P301L)#Kha/JlwsJ mice (Stock No: 015815), were obtained from Jackson Laboratory (Bar Harbor, ME, USA). The transgene has the Tet-responsive element (TRE or *tetO*) and mouse prion protein promoter sequences (PrP or *Prnp*) directing expression of the P301L mutant variant of human four-repeat microtubule-associated protein tau (4R0N tau_P301L_). The untranslated sequence from Prnp results in moderate levels of tauP301L expression in the brain before Tet-induction, but does not result in tauopathies. All animal work was approved by the Animal Care and Use Committee of the Nantong University (approved on July 20, 2012), according to the PHS Policy on Human Care and Use of Laboratory animals. Tau_P301L_ mice were bred and housed at 24 ± 1 °C, light-dark cycle of 12 hours/12 hours, and free access to food and water.

### Plasmids, proteins, and antibodies

Mammalian expression vectors pCI/CK1ε tagged with HA at the N-terminus or Flag at the C-terminus were generated by reverse transcription PCR from RNA isolated from normal human neuronal progenitor cells, cloned into pCI-neo vector and confirmed by DNA sequence analysis. pRK172 containing the largest isoform of human tau, pRK172/tau441, was kindly provided by Dr. Michel Goedert (Molecular Biology Unit, Medical Research Council, Cambridge, U.K.). pGEX-6P1/tau_441_, pCI/tau_441_ or pEGFP-N1/tau_441_ was constructed by PCR amplification from pRK172/tau_441_ and subcloning into pGEX-6P1 to express GST fusion proteins of Tau_441_, GST-tau441, into pCI-neo to express tau_441_, or into pEGFP-N1 to express GFP-tau_441_. pCI/SI9-LI10 containing a tau mini-gene, SI9-LI10, comprising tau exons 9, 10 and 11 and part of intron 9 and the full length of intron 10 was a gift from Dr. Jianhua Zhou of Massachusetts Medical School. For lentiviral gene transfer, enhanced GFP- and CK1ε expressing lentiviruses were obtained from GenePharma Company (Shanghai, China). The lentiviral vector coding for GFP was used as a control. The monoclonal anti-HA was bought from Sigma (St. Louis, MO, USA). The monoclonal anti-GST was bought from Novagen (Merck KGaA, Darmstadt, Germany). The monoclonal anti-3R-tau was from Upstate Biotechnology, Inc. (Lake Placid, NY). The polyclonal anti-GAPDH and anti-CK1ε were from Santa Cruz Biotechnology (Santa Cruz CA, USA). Monoclonal anti-human tau (43D) and polyclonal anti-tau (92e) were described previously^28^. Phosphorylation-dependent and site-specific tau antibodies against pS199-tau, pS396-tau, pS202-tau pT205-tau, pT212-tau, pS214-tau, pT217-tau, pS262-tau, and pS404-tau were purchased from Invitrogen (Carlshad, CA, USA). Monoclonal antibody PHF-1 that recognizes tau phosphorylated at Ser396/Ser404 was kindly provided by Dr. P. Davies of Albert Einstein College of Medicine, Bronx, NY, USA. Peroxidase-conjugated anti-mouse and anti-rabbit IgG were obtained from Jackson ImmunoResearch Laboratories (West Grove, PA, USA).

### Cell culture and transfection

HEK-293FT, N2a, SH-SY5Y and HeLa cells were maintained in Dulbecco’s modified Eagle’s medium (DMEM) supplemented with 10% fetal bovine serum (Invitrogen) at 37 °C. All transfections were performed in triplicate using FuGENE HD (Roche Diagnostics, Indianapolis, IN, USA) or Lipofactamine 2000 (Invitrogen) according to the manufacturer’s instructions.

### Quantitation of tau exon 10 splicing by reverse transcription-PCR (RT-PCR)

Total cellular RNA was isolated from cultured cells by using the RNeasy Mini Kit (Qiagen). One microgram of total RNA was used for first-strand cDNA synthesis with oligo(dT)15–18 by using the Omniscript reverse transcription kit (Qiagen). To measure the alternative splicing of Tau exon 10, PCR was performed by using PrimeSTAR^TM^ HS DNA polymerase (Takara Bio Inc., Otsu, Shiga, Japan) with primers 5′-GGTGTCCACTCCCAGTTCAA-3′ (forward) and 5′-CCCTGGTTTATGATGGATGTTGCCTAATGAG-3′ (reverse) at 98 °C for 3 min, 98 °C for 10 s, 68 °C for 40 s for 30 cycles, and 68 °C for 10 min for extension. The PCR products were resolved on 1.5% agarose gels and quantitated using the Molecular Imager system (Bio-Rad).

### Quantitative real-time RT-PCR (qRT-PCR)

Total cellular RNA was isolated from frontal cortical tissues using the RNeasy Mini Kit (Qiagen, GmbH, Germany) according to the manufacturer’s instructions. cDNAs were synthesized from 1 μg of total RNA using the Omniscript Reverse Transcription kit (Qiagen) and real-time PCR was performed using Brilliant II SYBR® Green QPCR Master Mix (Agilent Technologies, Inc. Santa Clara, CA) in the Agilent Mx3000p Real-Time PCR detection system under the condition: 95 °C for 10 min, 95 °C for 30 sec, and 60 °C for 1 min for 40 cycles. Each sample was analyzed in triplicate, and relative CK1ε gene expression was calculated using the comparative Ct method after normalization to the housekeeping gene GAPDH. Primers for CK1ε amplifications were forward 5′-CTACAAGATGATGCAGGGTGGCG and reverse 5′-AGGCTGAATTTGCGGGAACAGA. The primers for GAPDH were forward 5′-GGTGGTCTCCTCTGACTTCAACA and reverse 5′-GTTGCTGTAGCCAAATTCGTTGT.

### GST pull-down assay

Rat forebrain was homogenized in cold buffer (50 mM Tris-HCl, pH 7.4, 150 mM NaCl, 50 mM NaF, 1 mM Na_3_VO_4_, 0.1% Triton X-100, 2 mM EDTA, 1 mM PMSF, 10 μg/ml aprotinin, 10 μg/ml leupeptin, and 10 μg/ml pepstatin) and centrifuged at 15,000 xg for 10 min. The resulting supernatant was adjusted to 1 mg/ml protein concentration with the same buffer and used for GST pull-down assay.

GST and GST-tau_441_ were affinity-purified with glutathione-Sepharose without elution from the beads. The beads coupled with GST or GST-tau_441_ were incubated with above rat brain extract. After 2 hrs of incubation at 4 °C, the beads were washed with washing buffer (50 mM Tris-HCl, pH 7.4, 150 mM NaCl, and 1 mM DTT) six times and the bound proteins were eluted by boiling in Laemmli sample buffer and analyzed by Western blots.

### Co-immunoprecipitation

HEK-293FT cells were transfected with HA-tagged CK1ε construct, pCI/CK1ε, and pEGFP-N1-tau_441_ for 48 hrs, and then the cells were washed twice with phosphate buffered saline (PBS) and lysed by sonication in lysate buffer (50 mM Tris-HCl, pH 7.4, 150 mM NaCl, 50 mM NaF, 1 mM Na_3_VO_4_, 2 mM EDTA, 1 mM PMSF, 10 μg/ml aprotinin, 10 μg/ml leupeptin, and 10 μg/ml pepstatin). The cell lysate was centrifuged at 16,000 X g for 10 min and supernatant was incubated with anti-HA pre-conjugated protein G beads overnight at 4 °C. The beads were washed with lysate buffer twice and with TBS (50 mM Tris-HCl, pH 7.4, 150 mM NaCl) twice, and bound proteins were eluted by boiling in Laemmli sample buffer and analyzed by Western blots with the indicated primary antibodies.

### Immunostaining

Human postmortem brain tissues from normal and AD individuals were fixed and embedded in paraffin using standard methods. Slides with 6-µM sections from the paraffin-embedded specimens were deparaffinized in xylene and rehydration via transfer through graded alcohols. To unmask the antigenic epitope, the sections were treated with the antigen retrieval solution that contains 0.71 M citric acid and 0.1 M sodium citrate in a microwave oven for 2 min. Endogenous peroxidase was inactivated from the samples by incubation in 0.5% hydrogen peroxide in PBS for 10 min. Sections were then incubated with anti-CK1ε (Santa Cruz) at 4 °C overnight. After washing with TBS, horseradish peroxidase–conjugated anti-rabbit IgG antibody (Jackson ImmunoResearch Laboratories) was applied at room temperature for 2 hrs. The enzymatic reaction was developed in a freshly prepared solution of 0.01% hydrogen peroxide combined with 0.06% diaminobenzidine in PBS as a chromogen for horseradish peroxidase. The reaction was conducted simultaneously in every sample until a faint dark brown coloration was observed in the sections, and then stopped by washing with PBS. Prior to observation sections were mounted with DPX mounting solution.

SH-SY5Y or HeLa cells were plated onto coverslips in 24-well plates. HeLa cells co-transfected with pCI/CK1ε and pEGFP-N1-tau_441_ for two days or SH-SY5Y cells were washed with PBS and fixed with 4% paraformaldehyde in PBS for 30 min at room temperature. After washing with PBS, the cells were blocked with 10% goat serum in 0.2% Triton X-100/PBS for 2 hrs at 37 °C and incubated with primary antibodies: rabbit anti-HA (1:200), anti-CK1ε (4D7, 1:100), or anti-tau (R134d, 1:500) overnight at 4 °C. After washing and incubation with Alexa Fluor 555-conjugated or Oregon Green 488-conjugated secondary antibodies from goat (Life Technologies, Rockford, IL, USA)), the cells were washed extensively with PBS, and incubated with 5 mg/ml Hoechst 33342 or TO-PRO 3 iodide for 5 min at room temperature. The cells were washed with PBS, mounted with Fluoromount-G and visualized with a Leica TCS SP2 laser-scanning confocal microscope.

Mice were perfused with saline followed by 4% paraformaldehyde in PBS for 20–30 min. The brains were removed and post-fixed in 4% paraformaldehyde overnight. The following day all brains were cut in 40 µm coronal sections using a freezing microtome (Leica, Germany). Sections containing hippocampus were incubated in a blocking solution containing 5% normal goat serum and 0.2% Triton X-100 in TBS at 37 °C for 30 min, after which they were incubated overnight at 4 °C with primary antibodies. The sections were then incubated at room temperature for 1 hour with Cy3 goat anti-rabbit F(ab’)2 (Jackson ImmunoResearch Laboratories) (1:1000) in TBS. Immunostaining of pSer214-tau was carried out by conventional immunohistochemical protocol using SABC-Cy3 staining kit (Boster, Wuhan, China). The primary antibodies used included polyclonal phospho-tau antibodies anti-pS396, anti-pS199, anti-pS214 (1:200, Invitrogen). The immunostaining was visualized with a Leica TCSSP2 laser-scanning confocal microscope. The described protocol was also used for human brain tissue immunofluorescence.

### Transduction of cortical neurons with lentiviral vectors

Primary cortical neurons from embryonic day 17 (E17) Sprague Dawley rats were isolated. After isolation cells were resuspended in serum free Neurobasal medium supplemented with B27 (Invitrogen) containing penicillin (20 U/ml) and streptomycin (5 μg/ml), and plated on poly-L-lysine-coated 6-well plates. Rat embryonic cerebral cortical neurons were cultured for 3 days prior to infection with lentiviral vectors. The virus-containing medium was removed 24 hours later and replaced with fresh neurobasal medium.

### Intracerebral administration of viral vectors

At the age of 3 months, FVB-tau_P301L_ mice were anesthetized by an i.p. injection of chloral hydrate (400 mg/kg), and then mounted onto a stereotaxic apparatus (Stoelting Instruments, Wood Dale, IL, USA). 2 μl of lentivirus was injected into each hippocampus at a rate of 0.25 μl/min using a 25-gauge Hamilton syringe. Coordinates of injection site were anteroposterior −2.0 mm, mediolateral ± 2.5mm relative to the bregma, and dorsoventral −2.0 mm relative to the skull surface. The needle was left in place for an additional 4 min and then withdrawn. Control animals received vehicle only.

### Open field test

Mice were allowed to explore freely for 10 min in a square open plastic chamber (50 × 50 × 30 cm) and the apparatus was cleaned with 70% alcohol after testing of each mouse, during which total movements in the open field were recorded and analyzed by the Anymaze video tracking system (Stoelting Co., Wood Dale, IL, USA).

### Spontaneous alternation performance

Spontaneous alternation is mainly a hippocampus-dependent behavior for assessing working memory^52^. Male 10–12 mice of 13 months of age were used for the present study. Spontaneous alternation was assessed in a Y maze. Each mouse was placed at the center of the maze and allowed to move freely through it during an 8 min period. The number of arm entries from 2–8 mins was recorded and analyzed by the Anymaze video tracking system (Stoelting Co., Wood Dale, IL, USA). The percentage of alternation was calculated as (actual alternations/total entered-2) × 100. Spontaneous alternations are defined as consecutive triplets of different arm choices. The chance level for spontaneous alternations is 22%.

### Statistical Analyses

Data are presented as mean ± S.D. Data points were compared by Student’s t test for two-group comparison. For the analyses of the correlation between levels of CK1ε and tau phosphorylation, 3R-tau expression, Braak stage or Tangle score in human brains, the linear regression was performed, and the Spearman correlation coefficient *r* was calculated.
